# Visual and Auditory fMRI Paradigms for Presurgical Language Mapping: Convergent Validity and Relationship to Individual Variables

**DOI:** 10.1155/2019/6728120

**Published:** 2019-04-01

**Authors:** Antonina Omisade, Christopher B. O'Grady, Matthias H. Schmidt, John D. Fisk

**Affiliations:** ^1^Acquired Brain Injury (Epilepsy Program), Nova Scotia Health Authority, Halifax B3S 0H6, Canada; ^2^Department of Research, Nova Scotia Health Authority, Halifax B3S 0H6, Canada; ^3^Department of Diagnostic Radiology, Dalhousie University, Halifax B3H 4R2, Canada; ^4^Department of Psychiatry and Department of Medicine (Division of Geriatric Medicine), Dalhousie University, Halifax B3H 4R2, Canada; ^5^Seniors' Health, Nova Scotia Health Authority, Halifax B3H 2E2, Canada

## Abstract

Functional MRI (fMRI) has emerged as a safe alternative to invasive procedures for determining hemispheric language dominance prior to neurosurgery. Despite this, there are currently no standardized fMRI protocols that have been explored in healthy controls to determine the influence of individual patient variables on the results, which poses challenges in clinical interpretation of ambiguous findings in patient populations. In addition, most fMRI protocols are not suitable for individuals with visual or intellectual disabilities (IQ<70). In the current study, 61 healthy adults (ages: 18-74 years) completed two fMRI paradigms for language mapping. One paradigm used visually based stimuli and has shown good face validity to date in our center. The second paradigm used auditory stimuli presented at slowed speed and was designed for individuals with visual or cognitive dysfunction but has not yet been used clinically. The paradigms demonstrated 97% agreement in classifying individuals as left-hemisphere, right-hemisphere, and bilaterally dominant. Cases that were classified differently showed bilateral dominance in response to either paradigm. Dominance classification rates for right- and left-handed individuals were largely in keeping with published data. Within the left-handed group, IQ and education were positively correlated with laterality indices generated by both paradigms (r values range: 0.44-0.95, p<0.01), suggesting that individuals with higher IQ and formal education were more likely to be classified as left-hemisphere dominant in the current sample. This study will help improve clinical interpretation of language fMRI maps by identifying factors that might impact results (like IQ). It also offers an alternative paradigm to make this procedure more accessible to a broader range of patients. Future studies will replicate results with a sample of patients with epilepsy across a broad range of intellectual abilities.

## 1. Introduction

Functional magnetic resonance imaging (fMRI) has been used as an accessible and an effective adjunct noninvasive technique for presurgical language lateralization for people with focal epilepsy and brain tumors. Functional MRI detects brain regions activated during language tasks by measuring changes in oxygenated blood flow associated with increased metabolic activity (i.e., blood oxygen level dependent or “BOLD” response) [1]. Some have strongly advocated for fMRI as a replacement for invasive testing, like the Intracarotid Amobarbital Procedure (IAP) or etomidate speech and memory test (eSAM) [2, 3]. To date, fMRI in individuals with epilepsy has demonstrated over 90% concordance with these procedures. This, however, is true mainly in individuals with strong left-hemisphere language dominance [3]. In those with atypical language representation, such as bilateral or right-hemisphere dominance, concordance rates are substantially lower, ranging between 50 and 80% [4]. Currently, there are no fMRI protocols for language lateralization that have been standardized using healthy control populations prior to clinical application.

In Halifax, Nova Scotia, Canada, fMRI has been used routinely as an adjunct clinical technique for language lateralization prior to epilepsy surgery since 2015 and on a more experimental basis since at least 2010. Our paradigm is based on one used at the Toronto Western Hospital and involves a panel of language tasks that encompass receptive and expressive language functions [5, 6]. It also includes active control conditions that target visual and motor regions that are often activated in language tasks but that are not associated with verbal processing. The resulting fMRI maps are then reviewed by a neuropsychologist (A.O.), who is familiar with the patients' clinical history and neuropsychological findings. Laterality is determined by visual analysis of activation within the inferior frontal and temporal lobes, as well as in the cerebellum [7, 8] within the context of other clinical information like structural lesions on MRI, cognitive profile, and seizure semiology. Since 2015, we have administered this paradigm to 55 patients with epilepsy and/or tumors and have achieved excellent face validity and concordance with neuropsychological test findings. This paradigm reliably activates the main epicenters of the language network within the temporal lobe and the inferior frontal gyrus (Broca's area), as well as the cerebellum contralateral to the language dominant hemisphere. In most cases, the activation is clearly on the left and the findings are consistent with handedness and other clinical information including: side of epileptogenic lesion or tumor, seizure semiology, and type of disruption in language functions (if any) in the ictal and postictal periods. However, we have seen ambiguous or discrepant findings that we have been unable to fully interpret in approximately 25% of cases. In such cases, fMRI results may demonstrate activation in the hemisphere opposite to that expected on the basis of other clinical information, or fMRI activation may show varying degrees of bilateral activation or “crossed dominance”, where expressive and receptive language epicenters are located in different hemispheres. The reasons for these discrepancies are often unclear, but may depend on individual patient characteristics like level of cognitive abilities (IQ, reading, level, processing speed, etc.), handedness, sex, and age. To date, we have not explored the influence of individual characteristics on our fMRI findings in healthy controls, which may help elucidate some reasons for ambiguous clinical findings.

Most language paradigms are developed for individuals with normal or corrected to normal vision, average or near-average IQ, and normal literacy skills. Currently, an IQ score below 70 is considered a contraindication for fMRI because of the challenges that individuals with low IQ face in following written instructions and completing tasks as intended [9], which would result in difficult-to-interpret findings such as those we have noted in some of our clinical cases. We recently developed a second paradigm that uses auditory, rather than visual, stimuli delivered at a slower rate that may help limit the effects of visual impairment, intellectual limitations, verbal ability, and processing speed, on fMRI findings. However, the auditory paradigm has not yet been used clinically at our center. As such, it is unclear whether it would effectively tap the same language processes as our current paradigm that has been partially validated via clinical use.

Among additional individual factors that may influence fMRI results, handedness is the most well-known and utilized marker of hemispheric language dominance. Approximately 95% of right-handed individuals and 60-80% of left-handed individuals are left-hemisphere dominant for language [10]. The remaining 5% of right-handers and 20-40% of left-handers are either bilaterally dominant or right-hemisphere dominant [10]. Familial sinistrality has been linked to greater probability of atypical language representation in left-handers [11]. Furthermore, age-related changes in BOLD responses, including increased bilateral activation during semantic processes in sentence comprehension, have been documented in several studies [12]. These changes are thought to reflect a decline in gray matter volume in the normal ageing brain, which requires recruitment of additional cortical regions to maintain normal performance [12, 13]. Impact of sex on language dominance assessment has also been proposed, including the possibly greater propensity for bilateral language representation in females [14–17]. In order to accurately interpret fMRI findings within a clinical context, it is important to understand the potential influence of these factors on fMRI paradigms.

Despite good face validity of our current (“visual”) fMRI paradigm in our clinical epilepsy population, we have no data available from healthy controls to aid our interpretation of discordant or complex findings in the clinical population. We also do not know whether our novel, auditory paradigm will be as effective in tapping a broad spectrum of language abilities and helping identify and lateralize relevant brain regions.

The aim of this study was to establish convergent validity of the two fMRI paradigms, the visual paradigm and the novel auditory paradigm, in relation to each other, to blinded clinician determinations of laterality, and to published rates of language dominance with regard to handedness in the general population. We also aimed to better understand how the results are affected by individual patient variables like familial sinistrality, sex, age, education, and IQ [18]. Our hypotheses are as follows.

- The results of the two paradigms are expected to be highly correlated: both paradigms will produce very similar ratios of language-related activation (i.e., laterality indices or LIs) in the right versus left hemispheres for individual participants.
- We anticipate a high agreement between the two paradigms with regard to LI-based classification of participants into left, right, and bilateral language dominance groups and between LI-based and clinician-based classification of dominance.
- We expect that both paradigms will produce dominant classification findings that are in line with published proportions of left, right, and bilateral dominance for right- and left-handers.

## 2. Materials and Methods

This study was approved by the Nova Scotia Health Authority Research Ethics Board (NSHA REB).

### 2.1. Participants

Thirty-one right-handed and thirty left-handed healthy adults over the age of 18 were recruited from the community using the following inclusion criteria: (i) no history of brain injury or head injury with loss of consciousness, (ii) no history of any neurological disorder, (iii) no history of learning disabilities, (iv) no self-reported limitations in English literacy or fluency, (v) no claustrophobia, (vi) no uncorrected visual impairment that would preclude accurate perception of fMRI stimuli, (vii) any health condition that would preclude MRI scanning (e.g., metal implants, pace makers), and (viii) self-reported cognitive impairment that interferes with daily functioning. Handedness was initially determined by asking participants for their preferred writing hand and then confirmed using the Edinburgh Handedness Inventory [19]. Other demographic information including familial sinistrality, age, sex, and level of education were collected to help characterize the sample, but were not used as criteria for exclusion or inclusion. All participants provided written informed consent prior to study participation.

### 2.2. Procedures

**Figure 1:**
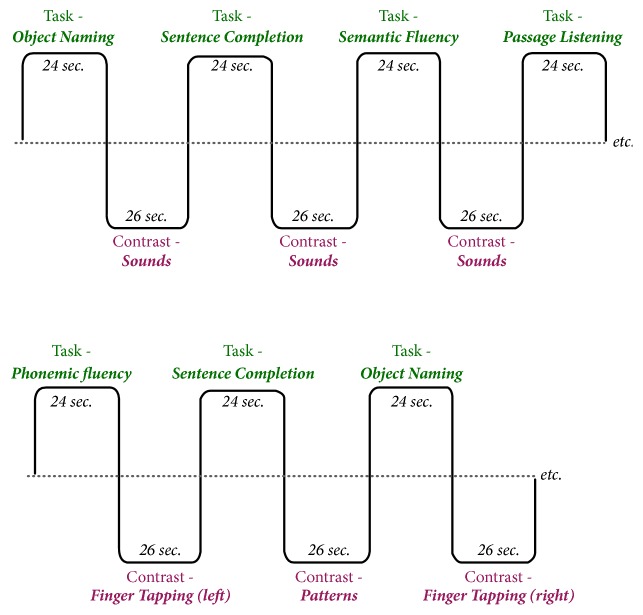
Diagrams of the auditory language paradigm (top) and of the visual language paradigm (bottom).

All participants completed the Wechsler Test of Adult Reading (WTAR) [20] to estimate full-scale IQ (FSIQ). MRI scanning was completed on the same day on a 3 Tesla GE MR750 Discovery scanner at the Halifax Infirmary. The scanning sessions lasted approximately 30 minutes and included one T1 structural image for precise anatomical localization of language areas and two functional sequences (one for each language paradigm) for each participant. The structural images were acquired using a spoiled gradient echo (FSPGR) sequence with TR/TE=5.7/2.1 msec, FOV = 22.4cm, FA = 12 degrees, 168 axial slices (1mm), in-plane voxel resolution = 1mm. Acquisition time was approximately 4.5 minutes. The functional images were acquired using the echo-planar pulse sequence with TR/TE=2000/25 msec, FOV=22 cm, FA=77 degrees, 48 axial slices (3mm), and in-plane voxel resolution =1.72 mm. Acquisition time for each functional scan was 7 minutes and 18 seconds. The two language paradigms were designed in accordance with general recommendations in the literature and recommendations by the American Society of Functional Neuroradiology [21]. The paradigms are depicted in Figure 1 and are briefly summarized here: (1) in visual paradigm, there are three language tasks (3 blocks per task, 24 seconds per block) and two control tasks (4 blocks per task, 24 seconds per block). Language tasks included silent word generation for specific letters, sentence completion, and naming to written description. All language stimuli were presented in written format via an overhead projector. The two control tasks included visual pattern discrimination and finger tapping. The control tasks were designed to activate areas of the brain that are frequently activated in performance of language tasks but are not language-specific (i.e., visual and motor regions). (2) In auditory paradigm there are four language tasks (2 blocks each, 24 seconds per block) and two control tasks (3 blocks each, 24 seconds per block). Language tasks consisted of silent word generation for specific letters, sentence completion, naming to description, and passive listening to a story passage. All language stimuli were presented auditorily through MRI-compatible headphones.

**Figure 2:**
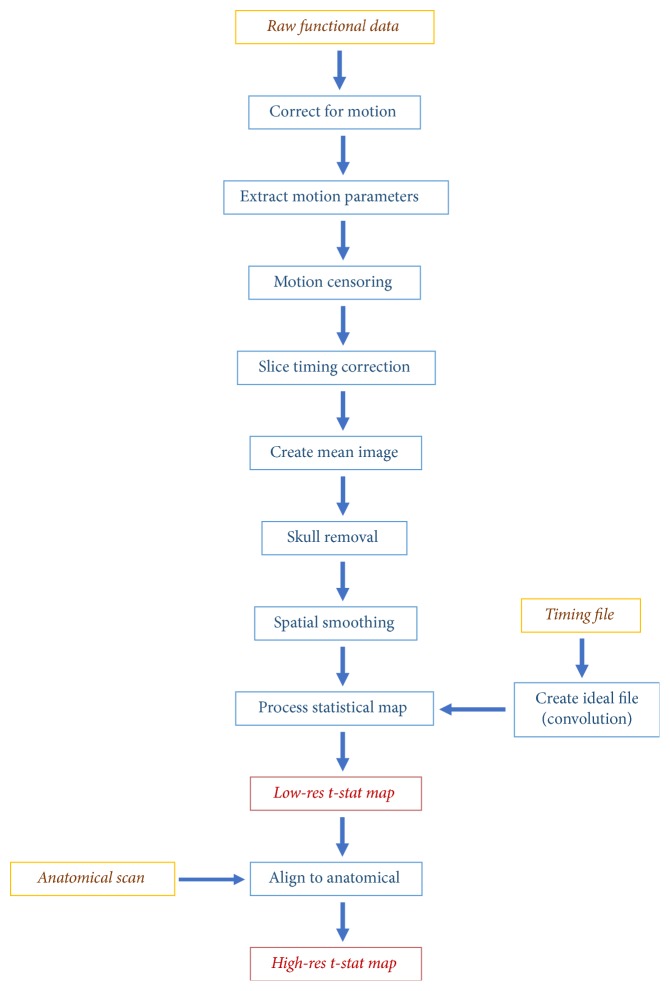
Processing pipeline used to analyze both fMRI paradigms.

**Figure 3:**
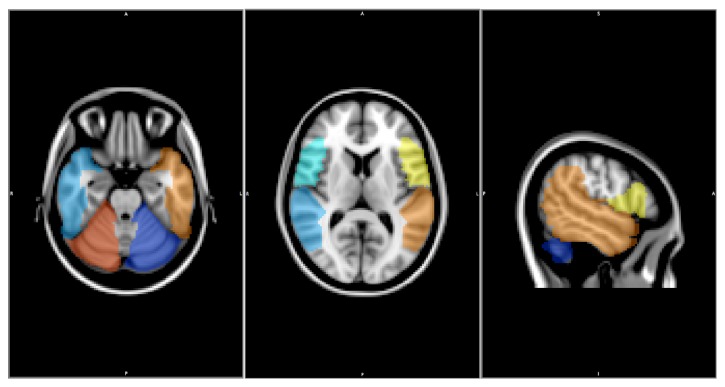
ROIs used to determine hemispheric language laterality. Yellow, orange, and red ROIs were used to calculate active voxels for left-hemisphere language dominance; blue, light blue, and light green ROIs were used to calculate active voxels for right-hemisphere language dominance.

Both paradigms were analyzed using the same processing pipeline that is demonstrated in Figure 2. Both functional and anatomical files were realigned to be in RPI coordinates to ensure correct orientation. The functional data was realigned to the first time series to correct for motion, and any voxels with large motion over a set threshold (0.3mm) were removed from further processing. Slice timing correction was completed and extra voxels outside the brain were removed with skull stripping before spatial smoothing to increase statistical power. The timing file was convolved with a gamma function representing the hemodynamic response and used with a brain mask of the functional data to calculate the statistical map, which was overlaid on the anatomical image. This statistical map indicated brain areas that show increased BOLD signal during language processing. The statistical maps were thresholded using the 98^th^ percentile of t-values indicating BOLD increase in response to language tasks [22] across the whole brain. Subsequent LI calculations and other statistical analyses were conducted using the voxels that fell about that threshold and that were located within the designated ROIs (i.e., temporal lobe, inferior frontal gyrus, inferior parietal lobes, and the cerebellum) (Figure 3) [7, 8, 23–25].

The voxels that fell above the threshold for significance were used to calculate individual LIs using

(1) $$\begin{array}{c} LI=\frac{\left(L-R\right)}{\left(L+R\right)} \end{array}$$

where L and R refer to number of activated voxels in the left and the right-hemisphere, respectively. LI cut-offs of 0.2 and -0.2 were selected to represent left and right-hemisphere language dominance, respectively [26–30]. LIs were used as the main method of classifying language dominance in this study to ensure consistency across participants and also in absence of additional clinical data in this healthy sample, like MRI abnormalities and cognitive profile, to aid with determination of language dominance. However, since review by a clinician is currently our standard method of determining language laterality, the maps were also reviewed by an experienced neuropsychologist (A.O.), who was blinded to participant identities.

Correlations between the LIs generated by the two paradigms for each participant, as well as associations between LIs and continuous variables like age, education, and IQ were examined using Pearson r. Agreement in categorical classification rates (right, left, and bilateral) between the paradigms and between paradigms and the clinician's ratings were completed using cross-tabulation. Associations between laterality classification categorical variables like sex and familial sinistrality were examined using Chi square analysis.

## 3. Results

Descriptive statistics for the entire sample and for the two handedness groups separately are presented in Table 1. There were no significant differences between the right- and the left-handers with regard to age, level of education, or estimated FSIQ.

**Figure 4:**
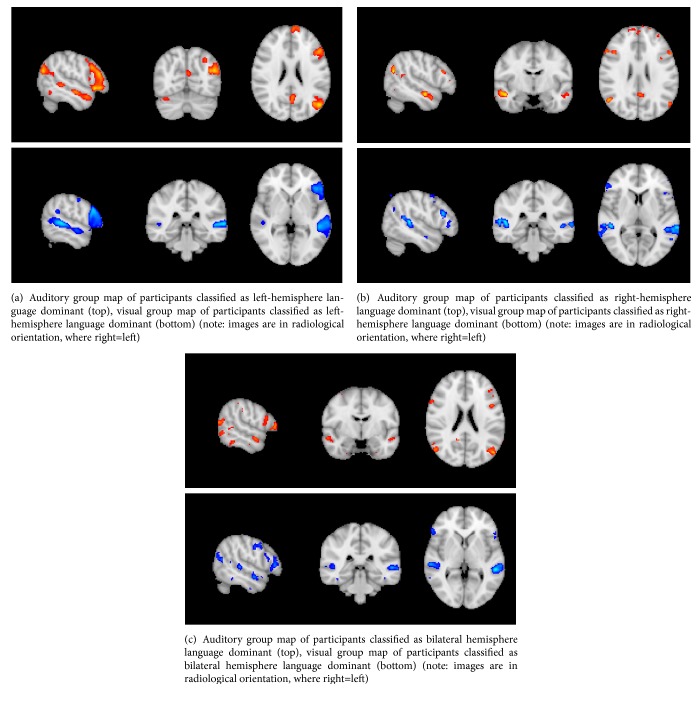


The laterality indices for both paradigms were highly correlated for the right- and left-handed groups (r=0.48, p<0.01 and r=0.93, p<0.01, respectively). Group maps can be seen in Figure 4. There was a 97% agreement between paradigms in classification of individuals as left, right, or bilaterally language dominant (Table 2). Discrepant classifications occurred in two cases. One right-handed participant was classified as bilaterally dominant by the visual paradigm, but left dominant by the auditory paradigm. One left-handed participant was classified as bilaterally dominant by the auditory paradigm, but left dominant by the visual paradigm.

Both paradigms also demonstrated a high rate of agreement between LI-based classification and classifications performed by a clinician blinded to participant identities. There was an 87% agreement between LI-based and clinician-based classifications for the visual paradigm (94% for right-handers, 77% for left-handers) and 85% agreement for the auditory paradigm (93% for right-handers, 80% for left-handers). Discordant cases were ones that were classified as bilaterally dominant using either classification method.

Both paradigms demonstrated left-hemisphere dominance for the majority of the sample. Using the visual paradigm, 30/31 (97%) right-handers were classified as left-hemisphere dominant, and one was classified as bilaterally dominant for language. Within the left-handed group, 26/30 (87%) individuals were classified as left-hemisphere dominant and three were classified as right-hemisphere dominant, and one was classified as bilaterally dominant. Using the auditory paradigm, 100% of right-handers were classified as left-hemisphere dominant. Within the left-handed group, 25/30 (83%) were classified as left-hemisphere dominant, three individuals were classified as right-hemisphere dominant, and two were classified as having bilateral dominance.

When the whole group was considered, there were significant positive correlations between IQ and education and auditory paradigm laterality indices (r=0.33, p<0.009, and r=0.26, p<0.04, respectively). These correlations were predominantly driven by the left-handed group, where LIs for both visual and auditory paradigms were strongly and significantly correlated with estimated IQ and level of education (r values range: 0.44-0.95, p<0.01). Neither age nor sex was associated either with LIs or with dominance classifications in either group. Familial sinistrality was not associated with LI scores or dominance classifications.

## 4. Discussion

In this study, we examined two paradigms for presurgical language mapping: the visual paradigm that is currently in routine clinical use in our center and that has demonstrated good face validity in patients with epilepsy and a novel auditory paradigm that was developed for individuals with visual impairment or cognitive limitations. We demonstrated that the results of the auditory paradigm with respect to individual LIs and determination of hemispheric language dominance in individual participants were in near-perfect agreement with our current paradigm. This indicates that our novel paradigm was as effective as our current “standard” paradigm in tapping into a range of language processes and identifying the likely dominant hemisphere. This was true for both right- and left-handed individuals and did not appear to be dependent on age or sex. As such, this paradigm can be used instead of our standard paradigm for individuals who may be unable to comply with the demands of the latter, at least for individuals of normal intelligence and without significant cognitive limitations.

Since this study included only healthy controls, the reproducibility of language laterality determined with fMRI in patients with epilepsy and/or epileptogenic lesions needs further examination. Epileptogenic foci can alter or disrupt language networks in variable and unpredictable ways. Furthermore, since this study did not include any individuals with IQs below 85, we cannot draw conclusions about the relationship between cognitive ability and fMRI findings based on this study alone. Though the auditory paradigm appears to work well with individuals of normal intelligence and is likely to be useful for individuals with visual impairments, additional research with a broader population is required to determine if it would work equally well for individuals with low IQs, for whom this paradigm was designed. In addition, our paradigms did not include a direct measure of behavioral responses to fMRI stimuli to ensure performance accuracy. Although this was not considered to be a major concern with healthy controls of average intellectual abilities who performed well on training tasks, this is likely to be an important factor to consider when administering these paradigms to neurologically compromised individuals.

We also demonstrated that, using both paradigms, the rate of left, right, and bilateral dominance differed between the right- and the left-handed groups, which would be expected from the literature. Within the right-handed group, 97% were classified as left-hemisphere dominant using the visual paradigm and 100% were classified as left dominant using the auditory paradigm; none were classified as right-hemisphere dominant for language. By contrast, the rates of left-hemisphere dominance were much lower within the left-handed group, with 87% and 83% for visual and auditory paradigms, respectively. Within the left-handed group, several individuals were also classified as right-hemisphere dominant using both paradigms.

The methods of determining handedness vary significantly across studies and clinical centers. This is particularly true for determining left-hand preference. While majority of right-handers tend to have very strong preference for using their right hand for all or most activities, left-hand preference tends to be more variable with many self-identified left-handers tending towards ambidexterity [10]. In addition, absolute determination of hemispheric dominance in healthy controls is impossible using invasive methods like IAP or eSAM testing or direct cortical stimulation. Thus, estimates vary widely in the literature depending on the methodology used. Due to a lack of “absolute” measures of handedness and language dominance in healthy controls, the reported range of left-hemisphere dominance in left-handers is wide, between 60 and 80% [10], which is generally in agreement with our findings and increases our confidence in the validity of our lateralization results. The true accuracy our classification results needs to be confirmed in a clinical sample of patients, who must undergo invasive testing as part of their standard presurgical clinical care.

Although these findings are encouraging regarding suitability of both paradigms for clinical use, they also highlight two potential factors that may affect the interpretation of individual clinical findings. First, our paradigms were less likely to be concordant with regard to hemispheric language dominance in individuals whose LIs were suggestive of bilateral representation. If an individual was identified as bilaterally dominant using either LIs or clinician ratings, there was a much greater chance of a disagreement in classification with either paradigm. Second, within the left-handed group, the LIs generated by both paradigms were positively correlated with education and estimated IQ. This may reflect a generally greater variability within the left-handed group with regard to LIs. However, it also suggests that this may be a significant factor to consider in interpreting individual clinical fMRI findings even in individuals with average or near-average IQ.

Since the results may be less reliable for individuals with lower IQ or educational attainment and for individuals with greater bilateral language activation on fMRI, these findings highlight the importance of using both LI-based and clinician-rated methods of determining hemispheric dominance, along with other clinical information.

## 5. Conclusions

This study is an important initial step in improving clinical interpretation of language fMRI maps by identifying factors that might impact results (like IQ) and also in offering an alternative paradigm to make this procedure more accessible to a broader range of patients. Future studies will expand on these findings and address the limitations of this research by recruiting controls and patients with epilepsy across a broad range of IQ, educational attainment, and cognitive/visual abilities. We will also correlate the findings with other clinical data collected from patients with epilepsy, such as seizure semiology, neuropsychological results, IAP/eSAM, or cortical stimulation findings, which would indicate language dominance.

## Figures and Tables

**Table 1:** Descriptive statistics of study participants.

Group	Age (+/- standard deviation)	Level of education (years)	FSIQ	Gender
Left-handed	37.5 (15.16)	16.5 (1.78)	115.47 (7.32)	20 female/10 male
Right-handed	40.94 (12.85)	16.32 (2.14)	112.84 (8.20)	15 female/16 male
Total	39.25 (14.0)	16.41 (1.95)	114.13 (7.83)	25 female/26 male

**Table 2:** Agreement between paradigms for classification of individuals as left, right, or bilaterally language dominant.

	Left dominant		Right dominant		Bilateral		
	Visual	Auditory	Visual	Auditory	Visual	Auditory	Agreement
Right handed	30/31 (97%)	31/31 (100%)	0/31 (0%)	0/31 (3%)	1/31 (3%)	0/31 (0%)	30/31 (97%)
Left handed	26/30 (87%)	25/30 (83%)	3/30 (10%)	3/30 (10%)	1/30 (3%)	2/30 (7%)	29/30 (97%)
Total	56/61 (92%)	56/61 (92%)	3/61 (5%)	3/61 (5%)	2/61 (3%)	3/61 (5%)	59/61 (97%)

## Data Availability

The functional imaging, cognitive, and demographic data used to support the findings of this study are restricted by the Nova Scotia Health Authority Research Ethics Board in order to protect patient privacy. Data are available from the corresponding author (Antonina Omisade; tonya.omisade@nshealth.ca) for researchers who meet the criteria for access to confidential data.
